# Cellulose Fabrics Functionalized with Sol–Gel Photocatalytic Coatings Based on Iron (III) Phthalocyanine Tetracarboxylic Acids–TiO_2_–Silica Hybrids

**DOI:** 10.3390/gels9110860

**Published:** 2023-10-30

**Authors:** Alina Raditoiu, Valentin Raditoiu, Monica Florentina Raduly, Augusta Raluca Gabor, Adriana Nicoleta Frone, Maria Grapin, Mihai Anastasescu

**Affiliations:** 1 National Research and Development Institute for Chemistry and Petrochemistry—ICECHIM, 202 Splaiul Independentei, 060021 Bucharest, Romania; alina.raditoiu@icechim.ro (A.R.); monica.raduly@icechim.ro (M.F.R.); raluca.gabor@icechim.ro (A.R.G.); adriana.frone@icechim.ro (A.N.F.); maria.grapin@icechim.ro (M.G.); 2 Institute of Physical Chemistry “Ilie Murgulescu”, 202 Splaiul Independentei, 060021 Bucharest, Romania; manastasescu@icf.ro

**Keywords:** photocatalyst, phthalocyanine, hybrid silica gels, cellulose, sol–gel coatings, xerogels, nanosols

## Abstract

Photocatalytic coatings are difficult to obtain on textile materials because of the sometimes contradictory properties that must be achieved. In order to obtain a high efficiency of a photocatalytic effect, the metal–oxide semiconductor must be found in the vicinity of the coating–air interface in order to come into direct contact with the contaminant species and allow light radiation access to its surface. Another necessary condition is related to the properties of the covering textile material as well as to the stability of the xerogel films to light and wet treatments. In this sense, we proposed a solution based on hybrid silica films generated by sol–gel processes, coatings that contain as a photocatalyst TiO_2_ sensitized with tetracarboxylic acid of iron (III) phthalocyanine (FeTCPc). The coatings were made by the pad–dry–cure process, using in the composition a bifunctional anchoring agent (3-glycidoxipropyltrimethoxysilane, GLYMO), a crosslinking agent (sodium tetraborate, BORAX), and a catalyst (N-methylimidazole, MIM) for the polymerization of epoxy groups. The photodegradation experiments performed on methylene blue (MB), utilized as a model contaminant, using LED or xenon arc as light sources, showed that the treatment with BORAX improves the resistance of the coatings to wet treatments but worsens their photocatalytic performances.

## 1. Introduction

In today’s world, environmental pollution poses a serious risk to human health and natural ecosystems, as well as a challenge to the scientific community in trying to adapt to the numerous negative effects of industrialization and progress. Even with scientists’ best efforts, water pollution’s harmful consequences grow exponentially and spread out. In order to eliminate the target contaminants despite their low concentrations, new technologies are thus continuously being proposed for remediation. Adsorption, membrane-based filter processes, oxidative processes, and other techniques can all be used to reduce pollution [1].

The advancement of advanced oxidation processes (POA) is one of the key directions in which water treatment technologies are evolving. As an alternative to waste incineration, which can cause significant issues owing to the release of harmful substances like polychlorinated dibenzodioxins and polychlorinated dibenzofurans into the environment, advanced oxidation techniques have gained importance [2]. POAs are oxidation procedures and technologies based on the in situ production of highly oxidizing radical and reactive species (peroxyl, hydroxyl organic radicals, molecular singlet oxygen, or superoxide radical anion), which interact with organic pollutants and result in their fragmentation [3]. Heterogeneous POAs necessitate the addition of appropriate solid semiconductors/catalysts (metal oxides, organometallic catalysts, or transition metal sulfides) [4] to trigger a photochemical process at the liquid/solid interface, where hazardous organic pollutants are oxidized and ultimately mineralized.

The solution studied by us is related to obtaining photocatalytic coatings on cellulosic fabrics, which could be used, in particular, in water depollution processes, functioning as photocatalytic adsorptive materials [5] for the decomposition of hazardous organic pollutants.

Photocatalytic coatings for different types of surfaces are widely investigated, and finishing formulations for textiles are studied in order to obtain self-cleaning photocatalytic surfaces [6]. This approach provides super-hydrophilic surfaces during irradiation due to the photocatalytic effect [7], which is the opposite of the highly hydrophobic or oleophobic finishing formulations applied as a well-established route in the textile industry to achieve repellent surfaces [8]. Therefore, the self-cleaning effect is due to the decomposition of contaminants by reactive species generated at the surface of the photocatalyst during irradiation with ultraviolet light [9].

The most important photocatalyst used in practice is anatase, which has a band gap of 3.2 eV and is able to oxidize and decompose organic molecules due to reactive species generated after the promotion of an electron from the valence band into the conduction band [10,11]. Since textile substrates are essentially organic materials and are sensitive to high temperatures, the processing temperatures are limited, and the photocatalyst should be obtained in a suitable crystalline form [12] before being deposited on the textile surfaces.

An important issue to overcome is related to the organic binders that are usually used to obtain coatings on textile materials. These types of binding materials can also become the subject of photocatalytic decomposition during irradiation [13,14]. Therefore, an important alternative is to use binders based on organic–inorganic hybrid systems [15,16], knowing that such systems provide better stability for photochemical decomposition of organic species [17].

The sol–gel technique is particularly useful for generating coating materials for textile finishing by dipping, spraying, or by using pad–dry–cure processes [18]. There is a lot of work in the field of silica nanosols obtained by hydrolysis–condensation and deposited onto the surface of textile materials for coloring [19] or as protective coatings, hydrophobic finishing materials [20], reinforcing coatings for technical textiles, or as fire-retarding materials. Intense concerns in this direction are based on the general properties of nanosols, which are able to generate hybrid organic–inorganic networks by entrapping or linking organic species into the inorganic oxide hosts and allow for tailoring the properties of the coated surfaces [21].

An important drawback of this type of material is usually related to the low flexibility of the film-forming materials due to the high content of inorganic compounds [22]. In this sense, concerns about reducing the thickness of the films and simultaneously obtaining highly adhesive and crack-free coatings on the textile surfaces have solved, in part, this issue [23]. Moreover, modification of the inorganic host with flexible organic crosslinking groups will result in a lower rigidity of the matrix and crack formation can be at least partially overcome [24].

Regarding the presented data, we proposed to address the issue of immobilization on a cellulosic support of a photocatalyst, earlier developed by us [25], which uses the visible light of the spectrum in the process of photodecomposition of organic contaminants. The proposed immobilization process is innovative by obtaining organic–inorganic hybrid silica gel coatings, which chemically react with the textile support and ensure the chemical and photochemical stability of the coating, at the same time ensuring a good photocatalytic effectiveness by exposure to natural (simulated in accelerated mode) or artificial (LED) light. The novelty of the method is based on the use of a bifunctional anchoring agent 3-glycidoxipropyltrimethoxysilane (GLYMO), which can develop organic and inorganic polymeric structures at each end of the molecule. By varying the working conditions and additives, coatings can be coupled, on the one hand, with the textile support and on the other hand, with the photocatalyst.

## 2. Results and Discussion

**Scheme 1:**
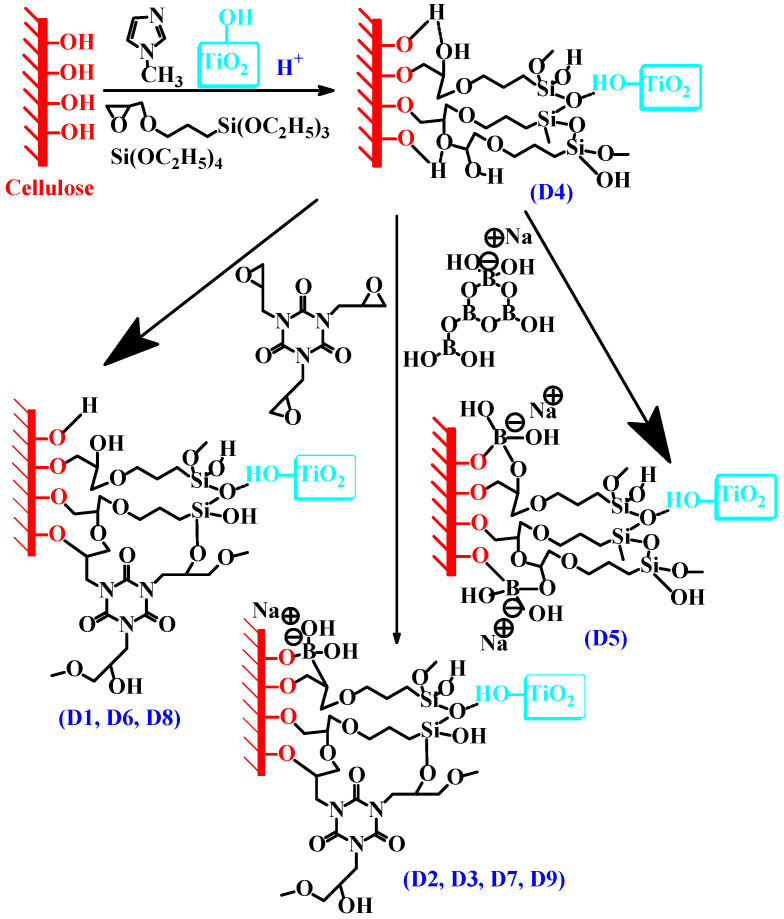
Interactions between components of photocatalytic hybrid silica gel coatings and cellulose.

One of the main problems of the hybrid coatings obtained with nanosols on textile substrates is represented by the stiffening of the obtained structures. In order to diminish this impediment, many experimental studies were conducted [26], including some developed by us, as previously reported [19]. The most important result of the conducted studies was the fact that the presence of the 3-glycidoxipropyltrimethoxysilane (GLYMO) bifunctional network modifier in the composition of the nanosol leads to obtaining the most flexible hybrid structures of organically modified silica, compatible with cellulosic textile fabrics. Starting from this finding, a catalyst (N-methylimidazole (MIM)) was introduced for the homopolymerization of glycidyl groups so that the generation of type II hybrid polymer structures (connected by covalent bonds) takes place (Scheme S1). Having as a starting point a nanosol composition optimized by us for applications on cellulosic textile supports, a photocatalyst (TiO_2_-FeTCPc) based on TiO_2_ sensitized with iron (III) phthalocyanine tetracarboxylic acid (FeTCPc), dispersed in isopropyl alcohol, was added to the composition under ultrasonic-assisted stirring. Hybrid silica gel structures were obtained by acid catalysis that accommodate TiO_2_-FeTCPc nanoparticles and bind to the cellulose structure through functional groups, as can be seen in Scheme 1.

However, the bonds formed between the hybrid silica gel coatings and the cellulosic material are of the C-O-Si type and less C-O-C in the case of the bifunctional agent with glycidyl groups. Ether groups are more stable to hydrolysis, especially in a neutral or alkaline medium, while due to the polarization of the C-O-Si bond, this is of the same nature as those of the silane residues that hydrolyze in an aqueous medium regardless of pH. This shortcoming causes such silica xerogel coatings to become unstable and ultimately leads to the detachment of the coating from the cellulose surface, either by washing, by the action of atmospheric agents, or by mechano-chemical actions.

In order to improve the bond stability of the hybrid silica gel coatings with the cellulosic support, two types of anchoring and crosslinking agents were used: tris(2,3-epoxypropyl) isocyanurate (TGIC) and sodium tetraborate (BORAX). These compounds are well known for the crosslinking reactions they give with alcohol groups [27,28], which would, in principle, create a stronger bond between the hybrid silica gel network and the textile support.

According to the observations made during the preparation of the nanosols, the generation of hydroxyl groups during the polymerization of the epoxy groups in GLYMO leads to compatibility of the film-forming material with the cellulosic support through the formation of hydrogen bonds that will ensure high adhesion forces at the textile support-hybrid coating interface. Moreover, the silanol groups from the hydrolysis of silanes will also contribute fully, together with the hydroxyl groups on the titanium dioxide surface, to the formation of these types of bonds. Furthermore, the carboxyl groups in the phthalocyanine sensitizer will be used as capping groups for anchoring it to the titanium dioxide surface, giving the particles a less hydrophilic character [29]. This will lead, during drying, to the migration of these photocatalytic particles to the coating–air interface, which will be involved in the good behavior of these xerogel films in photodegradation processes.

In principle, all of the materials added to the nanosol should promote the formation of ether bonds and the disappearance of residual hydroxyl groups following the thermal treatment carried out in the final stage in order to stabilize the silica gel coatings on the textile surface. As shown in Table 1, due to the low solubility in nanosols, a treatment with BORAX was conducted, and in some cases, at a later stage (subseq. stage) of the textile’s impregnation with nanosols, before the thermal treatment.

### 2.1. Structural Characterization by FTIR Spectroscopy

The structural characteristics of cellulose are visible in the infrared absorption spectra at 3334, 3291 cm^−1^ caused by hydroxyl stretching vibrations and at 2898, 2873 cm^−1^ for methylene symmetric and asymmetric stretching vibrations belonging to the linear chains of glucopyranosic units. The band specific to the bending vibration of water molecules contained in the fibers was found to be at 1630 cm^−1^. Other significant peaks can be found at 1428 cm^−1^, which are methylene wagging vibrations, and at 1314 cm^−1^, which are C-OH stretching vibrations. Bands corresponding to CH bending vibration at 1280 cm^−1^ and OH in-plane bending at 1244 cm^−1^ may also be observed. However, the most significant bands may be observed at 1159 cm^−1^ due to the asymmetrical bridge CO-C stretching vibration, at 1107 cm^−1^ from asymmetrical in-plane ring stretching, and at 1053, 1028, and 1000 cm^−1^ bands from the C-O stretching mode [30]. Another significant band was found at 710 cm^−1^ and is caused by the rocking vibrations of the CH_2_ group. In the range 700–400 cm^−1^, there are additional bands that correspond to OH out-of-plane deformations and vibrations of methylene groups.

**Figure 1:**
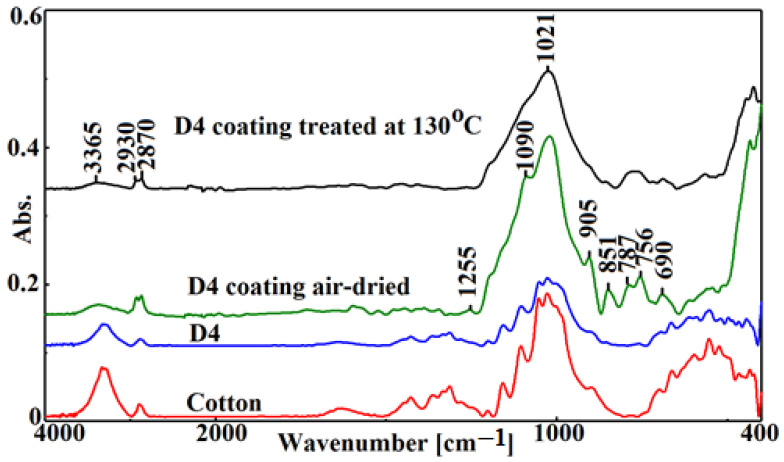
Comparisons among FTIR spectra of cotton (support), D4 (coated support), and D4 coated material air-dried and thermally treated at 130 °C.

Regarding the analyzed samples obtained by covering the cellulose with nanosols, they show many of the absorption bands characteristic of the xerogel coatings in the IR range, overlapping with those of the cellulose. That is why the spectra of the coating materials before and after the heat treatment were also recorded, the results being presented in Figure 1.

The two IR spectra of the coating material actually show us the areas of interest from the structural point of view of the xerogel coating, the exception being their connections with the textile support, as well as the chemical transformations undergone during the heat treatment. The FTIR spectrum of the D4 coating shows the stretching vibrations of hydroxyl groups as a large band centered at 3365 cm^−1^. The absorption band shrinks very little after the thermal treatment due to the existence of hydrogen bonds between the alcoholic and silanol groups in the structure. At 2930 and 2870 cm^−1^ are found characteristic bands of methylene symmetric and asymmetric stretching vibrations belonging to GLYMO units. Thermally treated D4 coating no longer presents the band at 1630 cm^−1^ of the water bending vibration or the large and asymmetrical band with a maximum at 1255 cm^−1^, which is associated with epoxy ring breathing vibration and disappeared during the heating, either due to hydrolysis or condensation with alcohol groups. It can be seen that the bands at 1090, 1021, and 905 cm^−1^ should be related to silane hydrolysis–condensation reactions because they are characteristic of Si-O-Si, Si-O-C, and Si-OH stretching vibrations [31]. Overlapped with the Si-OH stretching vibration is found the C-O stretching vibration of the epoxy ring. The bands at 787 and 756 cm^−1^ can be assigned to Si-O-C bending vibrations, and they have become equal in intensity and diminished in intensity, forming a very broad peak, during the thermal treatment.

Sample D4 was also investigated by FTIR after LED and xenon light irradiation (Figure S1). Differences between the FTIR spectra are minor; however, the sample irradiated with the xenon lamp presented an increased intensity of the band at 1630 cm^−1^ due to a higher content of water generated during photocatalysis and the appearance of a new band at 1234 cm^−1^, probably due to OH in plane bending, which is formed during epoxy group hydrolysis. This finding will be useful later in order to explain the thermo-mechanical behavior of the sample.

### 2.2. XRD Analysis

**Figure 2:**
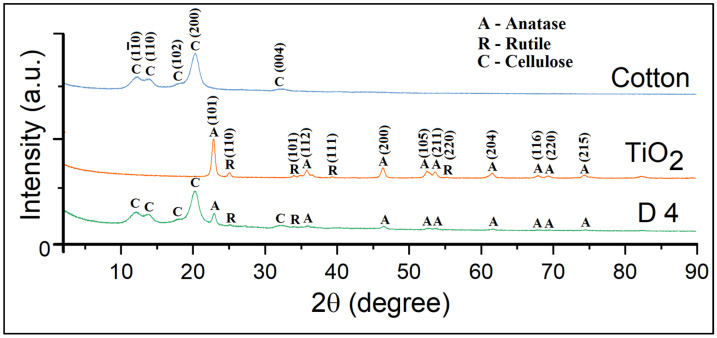
X-ray diffraction pattern of the D4 sample.

X-ray diffraction (XRD) analysis of the coated textile materials shows the presence of the characteristic lines of cellulose, as well as of titanium dioxide. It is obvious that due to the fact that the silica hybrid film is very thin, there is no characteristic band of the amorphous polymer. The XRD diffraction pattern showed the presence of the anatase phase (JCPDS file 00-021-1272 of the ICDD database), and in addition to anatase, the rutile phase was also found (JCPDS file 01-070-7347 of the ICDD database). The occurrence of the amorphous region in nano-celluloses was linked to the presence of the (004) lattice plane, which was brought about by the distinctive peak at 2θ = 32°. These planes are in accordance with the specifications of JCPDS Card No. 00-056-1718 in relation to the cellulose Ib data, as can be seen from Figure 2.

### 2.3. Surface Analysis by Scanning Electron Microscopy

**Figure 3:**
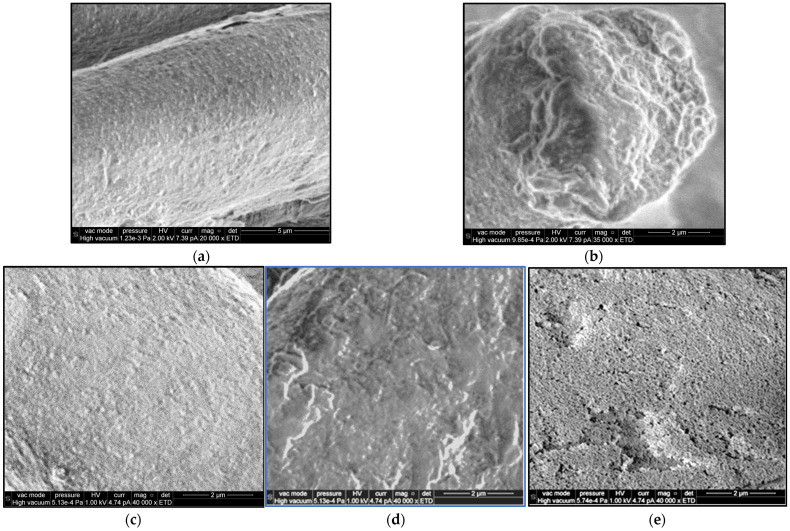
SEM images of (**a**) D4 coating, (**b**) D4 coating at the end of one cotton thread, (**c**) D4 initial, (**d**) D4 after exposure to LED light, and (**e**) D4 after exposure to arc xenon light.

Scanning electron microscopy was used as a tool in order to investigate the surface of the coated textile materials. The surface of the fabric appears to be uniformly covered by the photocatalytic xerogel film, as can be seen from Figure 3a. At one of the ends of the cotton thread, several layers of filmogen material can be observed in Figure 3b, as in the case of successive layers of “volcanic lava”. The surface shows a small roughness, most likely due to the TiO_2_-FeTCPc nanoparticles.

By means of SEM imaging, the behavior of the surfaces of the photocatalytic silica gel coatings upon exposure to LED light and xenon arc was also studied. In the first case, the lack of UV radiation from the LED light spectrum means that the surface does not significantly change its appearance, which translates into a good resistance to exposure to visible light. In the second case, a transformation of the surface is observed through the appearance of an accentuated roughness. It is very possible that, in this case, the organic part of the hybrid xerogel coating will degrade, as can be observed in Figure 3e.

A very high crosslinking degree is also possible with the formation of a very brittle film that cracks when handled, with some cracks in the spongy film being visible in Figure 3e. Moreover, the surface roughness increases after exposure to UV light for the same reasons as those previously mentioned. This will be verified through further investigations of the results obtained through the present study.

According to the EDX analysis, applied to determine the elemental composition on the surface of the cellulosic support coated with the composition D4, a content of 25.43% C, 70.83% O, 1.44% Si, and 2.06% Ti was determined, the difference being mainly represented by Ca 0.19% (from the cell wall of cotton and 0.05% chlorine from the chemical bleaching process of the fabric).

**Figure 4:**
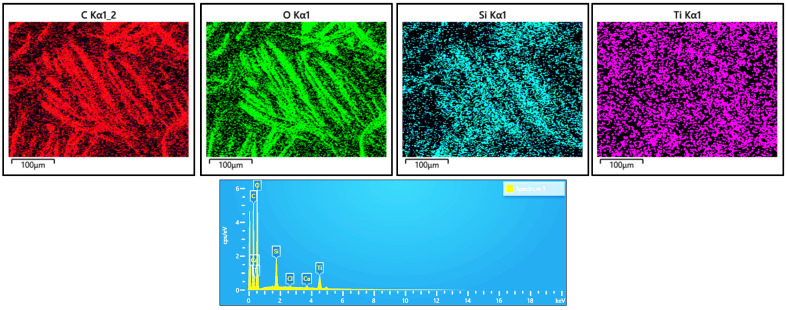
D4 (before exposure to light) elemental mapping by energy-dispersive X-ray spectroscopy (EDX).

Qualitatively, as it can be seen from Figure 4, the elemental distribution suggests the uniformity of the coating, and the disappearance of the fibrous structure in the case of titanium, which seems distributed over the entire surface, suggests that it is found at the interface of the hybrid silica gel coating with air and not in its interior.

### 2.4. Topography of the Coatings by Atomic Force Microscopy (AFM)

**Figure 5:**
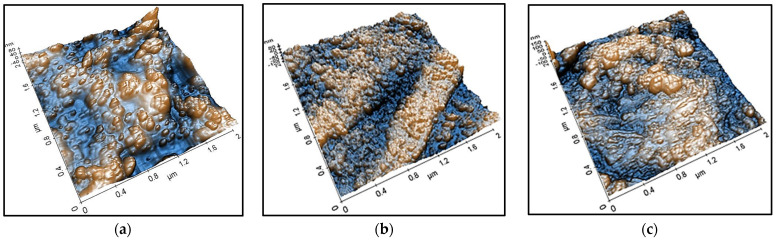
AFM images of (**a**) D4 coating initial, (**b**) D4 coating after exposure to LED light, and (**c**) D4 coating after exposure to arc xenon light.

By comparing the topographical data of coatings such as the one in the D4 case, shown in Figure 5a, with the D8 coating, there is an increase in the roughness Rq from a value of 19.64 nm in the first case to a value of 30.54 nm in the second case. This is explained by the crosslinking of the hybrid silica gel coating through the participation of TGIC, GLYMO, and MIM, which probably leads to a more pronounced migration of TiO_2_-FeTCPc toward the interfaces. This is demonstrated by the fact that in the case of D5, the use of BORAX leads to a lower roughness of only 14.97 nm.

Regarding the exposure to LED light or xenon arc, in the case of D4 as it can be seen from Figure 5b and Figure 5c respectively, an increase in roughness is found from the initial 19.64 nm to 23.17 nm after exposure to LED light or to 26.60 nm after exposure to xenon arc light. This confirms the fact that the crosslinking of organic residues is responsible for these transformations, except that in this case, it is about photochemistry and initiation through the charges generated by the TiO_2_-FeTCPc, especially when the exciting light also contains UV radiation.

### 2.5. Water Contact Angle Measurements

**Figure 6:**
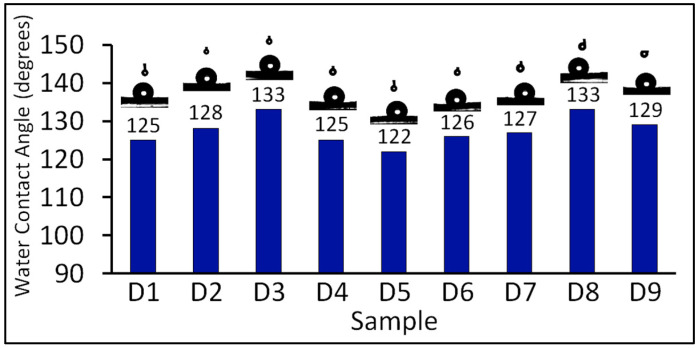
Water contact angle of the coated fabrics.

While analyzing the contact angle of water with the cellulose support covered with the different photocatalytic compositions studied by us, their hydrophobic character is noted, as can be seen in Figure 6.

This can be due to both the roughness of the xerogel films and the fact that the TiO_2_ nanoparticles are sensitized with the very hydrophobic FePcTC. Crosslinking ensures a more hydrophobic character, and therefore, in cases where BORAX was not used, low water contact angles are obtained. However, the maximum difference between the contact angles is small, only 11°, which shows that the advanced crosslinking attempts do not significantly change their general hydrophobic character. This observation is in full agreement with those previously obtained from SEM and AFM analysis.

### 2.6. Textural Properties

The adsorption isotherms obtained correspond to type VI according to the IUPAC classification and represent multilayer adsorption. Following the calculations by the BJH method, the low values of the surface area, as seen from Table 2, suggest that during the degassing process, irreversible changes occur in the structure of the cotton fibers due to the elimination of water molecules. Thus, the dehydration of the cotton fibers favors the formation of intercalated hydrogen bonds, and the measurement of the specific surface area of the fibers is mainly performed on the outer surface.

The low value of the pore volume compared to those obtained for other types of cellulose membranes [32] may be due to the collapse (modification) of the cellulose structure following the degassing/drying process. Thus, as specified in the literature, the main adsorption mechanism for cotton fabrics is represented by capillary adsorption. It is obvious that the textural properties of the photocatalytic textile materials will not influence the kinetic processes of photocatalytic decomposition of MB. However, the pore size is sufficiently high to accommodate an MB molecule, which has a length of 1.44 nm and a width of approximately 0.95 nm [33].

### 2.7. Sensitizer Stability to Light by CIEL*a*b* Color Measurements

The color differences in the CIEL*a*b* system are obtained from the L, a*, b* parameters based on the processing of the diffuse reflection spectra in the visible range. The total color difference (CIEL*a*b*) was calculated with Formula (1):

∆Eab = ((∆L*)^2^ + (∆a*)^2^ + (∆b*)^2^)^1/2^ (1)

where L* is the lightness parameter (from black to white), a* is the color deviation from green to red, and b* is the color deviation from blue to yellow [34].

It is known that silica gel coatings improve the light resistance of organic dyes and textile materials [35]. Practically, when organic dyes are encapsulated in such systems, they are more difficult to degrade under the action of ultraviolet radiation. Based on this fact, we can correlate the results obtained by calculating CIEL*a*b* color differences of fabric samples covered with hybrid silica with TiO_2_-FeTCPc after exposure to arc xenon light. The greater the color differences, the more the sensitizer is exposed to ultraviolet radiation and, as a consequence, the TiO_2_-FeTCPc is closer to the interface of the xerogel coating with air.

**Figure 7:**
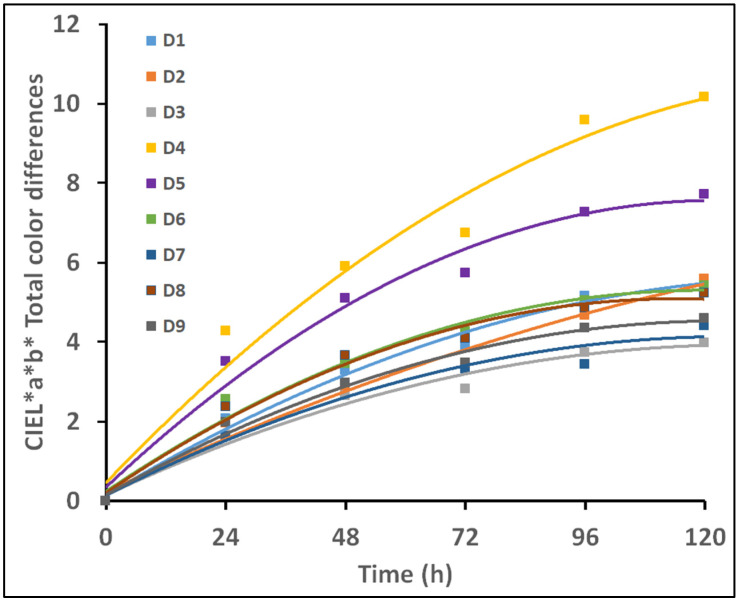
CIEL*a*b* total color differences of coated fabrics during exposure to arc xenon light.

From the measurements, it is obvious that D4 has nanoparticles of the TiO_2_-FeTCPc closest to the surface, which will make it highly efficient. D8 belongs to the same category, which will have a slightly lower performance, as can be seen in Figure 7. These qualitative estimates will be verified by the following degradation experiments.

### 2.8. Photocatalytic Efficiency

The textile materials coated with photocatalytic xerogel films were tested regarding the efficiency of MB decomposition, used as a contaminant. The exposure was made both in xenon arc light, which simulates natural light in a higher intensity mode, and with LED irradiation. The xenon arc light also contains UV radiation, which makes the decomposition process of the contaminant take place very quickly. Exposure to LED light, which has only the visible spectrum, shows a very high efficiency in the case of some of the sol–gel coatings, which leads to the conclusion that the organic sensitizer integrates very well into the analyzed photocatalytic systems. According to experimental evidence, P25’s surface-generated charges are mostly responsible for the photocatalytic activity’s apparent increase under UV light. The outcomes are consistent with two different types of mechanisms driving the photocatalytic activity under UV–Vis light produced by a xenon lamp and under LED light illumination. According to prior research, under UV light, holes in the valence band (VB) combine with adsorbed water to produce hydroxyl radicals, which oxidize contaminants, whereas electrons photogenerated in the conduction band (CB) react with oxygen to make superoxide radicals or hydroperoxides [36]. The oxidized version of the dye is produced in the second scenario, when the sensitizer is stimulated and electrons are injected into the TiO_2_’s CB, but the VB is left unaltered. Regeneration is accomplished by taking one electron from an organic species, which is then broken down [37].

The mechanism of TiO_2_-FeTCPc excitation at LED light illumination is presented in Figure S3, while the mechanism of MB photocatalytic decomposition was proposed and sustained by GC-MS analysis [38].

**Figure 8:**
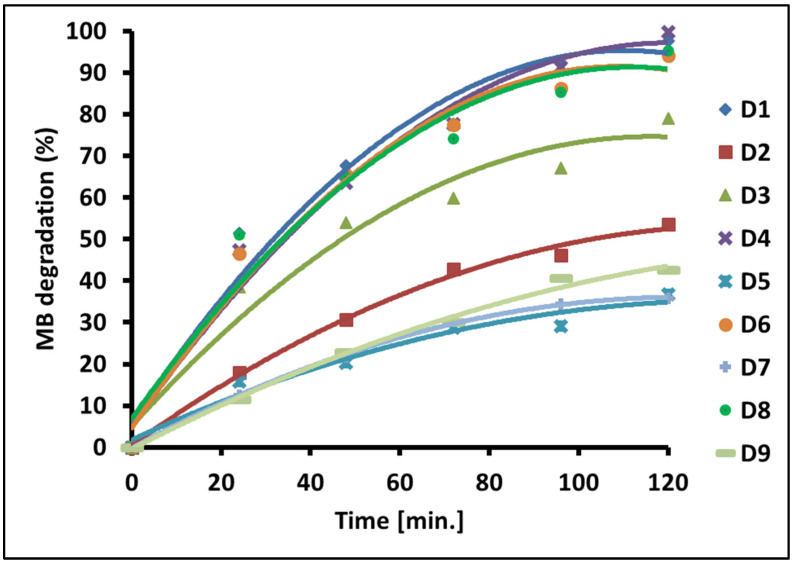
MB degradation efficiency during the exposure of photocatalytic fabrics to arc xenon light.

As can be seen from Figure 8, there are significant differences in the efficiency of the same type of TiO_2_-FeTCPc depending on the components of the type of sol–gel coating material and the subsequent treatments to which the textile material is subjected after impregnation with nanosols. Thus, there is a group of four coatings generally made up of nanosols containing only MIM and/or TGIC, such as D1, D4, D6 and D8. They have an MB photodecomposition efficiency between 91–98%, the highest being recorded in the case of D4.

All of the cotton samples covered with photocatalytic nanosol that were subjected to crosslinking with BORAX show a low efficiency in the MB photodegradation process, probably due to the formation of bonds on the surface of the TiO_2_-FeTCPc, which has as a consequence the inactivation of its surface. These are D2, D5, D7, and D9, which have an efficiency between 36–54%. A particular situation is encountered in the case of D3, which had BORAX in the composition of the nanosol and which probably did not interact to such a great extent with the TiO_2_ surface. This is the only way to explain the efficiency of the photocatalytic decomposition of MB of 75% recorded under the same conditions.

**Figure 9:**
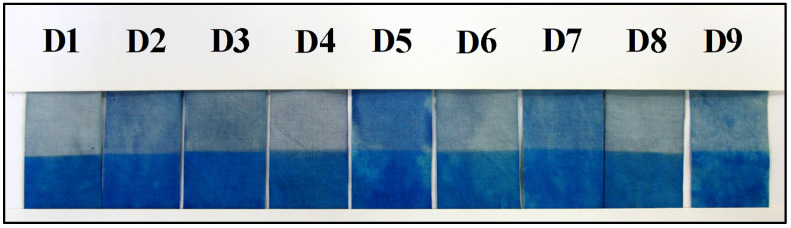
Cellulose fabrics coated with photocatalytic films at the end of the exposure to LED light.

In the case of exposure to LED light, MB decomposition takes place in the same way, but it is more pronounced in the case of D1, which in this case becomes the most efficient, with the following positions being occupied by D4, D8 and D6, as can be seen from Figure 9. In this case, the duration of the MB discoloration process is 30 h.

**Figure 10:**
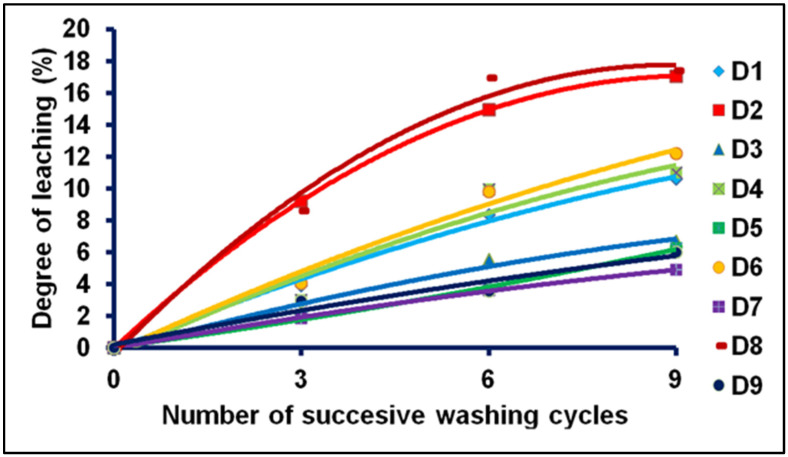
Degree of leaching during successive washing cycles.

Regarding the loss of the coating material through washing, it is noted that there are three types of photocatalytic xerogel coatings, depending on the resistance to washing, as can be seen from Figure 10. The sol–gel coatings with the worst resistance to the action of water are those obtained with nanosols D2 and D8, which after nine washing cycles lose almost 18% of the photocatalytic material. The second group is made up of the xerogel films obtained with D1, D4, and D6 nanosols, which after the same number of washing cycles lose up to 12% of the photocatalytic material. It should be noted that after three washing cycles, the leaching degree is at about the same level as that obtained in the case of the third category (the most resistant), i.e., up to 4%.

The most resistant photocatalytic silica gel coatings are obtained with nanosols D3, D5, D7, and D9. In order to be able to elucidate the causes that lead to such behavior, we must analyze the data in Table 1, which shows the presence or absence of the three components selected as necessary to strengthen the connection between the coating and the textile support, namely MIM, which is necessary for the homopolymerization of epoxy groups, TGIC, which is able to form stable bonds between the alcoholic groups of the substrate and of the hybrid network, and the BORAX used as a crosslinking agent for alcoholic groups.

As expected, the xerogel coatings that have BORAX in their composition are the most resistant, with the exception of D2, where the lack of MIM leads to the impossibility of homopolymerization of the epoxy group in GLYMO, which has, as a consequence, a lack of alcohol groups in the structure of the hybrid silica xerogel with direct consequences for the formation of stable C-O-C bonds with alcohol groups in cellulose. The film obtained with the composition D2 is in the category of the least resistant to washing, together with D8, which, even if it has MIM and TGIC in its composition, was not treated with BORAX, which thus proves its decisive importance in the film’s resistance to washing. The coatings without BORAX treatment carried out after impregnation with nanosols show an intermediate behavior and are found in group II of washing resistance. It is obvious that during repeated washings, the intercalation of water molecules in the structure of the hybrid silica gel occurs gradually, being delayed by the polymers generated from the epoxy groups, which maintains the level of removal by washing the photocatalytic coating material within acceptable limits and very close to the one obtained with the most resistant silica gel coatings until the third washing cycle. The arrival of water molecules in the vicinity of the Si-O-C bonds formed at the hybrid silica–cellulosic support interface leads to the occurrence of hydrolysis with a more pronounced detachment of the xerogel films from the textile support, which translates into the loss of more than 10% of the photocatalytic film recorded at the ninth washing cycle.

The factors that contribute to the differences in photocatalytic efficiency are related to the inactivation of some parts of the photocatalyst surface through interactions with components of the nanosols or the subsequent treatments with crosslinking agents; the adhesion of the xerogel coatings to the textile support and maintaining the integrity of the film during operation; contamination of the film surface with compounds resistant to photodegradation; and prevention of the access of light to the surface of the photocatalyst. These factors lead, from a practical point of view, to inefficiency by extending the decontamination time and limiting decontamination to a certain threshold (usually very low), and to frequent replacement of materials damaged by the action of water, inefficiency when operating in visible light and hence the impossibility of use in indoor applications together with LED light sources, and specificity for different types of polluting agents.

Water resistance is very important from the perspective of using the photocatalytic materials studied by us in applications related to the decontamination of water sources. Erosion of the surface under the action of running water, weak adhesion of the sol–gel coating to the substrate, and the instability of some chemical bonds in aqueous environments will all lead to the removal of the photoactive layer with an immediate effect on the efficiency of the photocatalytic process. Even in the case of technical textiles used for panels, banners, canopies, tents, and shelters, it is necessary that these materials have a high resistance to the weather (the action of rainwater) when used in the outdoor environment. Maintenance cleaning of surfaces exposed to mud, bird or animal droppings, or other materials that prevent light from producing the photocatalytic effect is also necessary.

### 2.9. Dynamic Mechanical Analysis (DMA)

In order to improve the adhesion of the silica gel coating to the surface of the cotton fabrics, TGIC was added to the nanosol composition. Interactions between components of the xerogel coating material and forces established between the coatings and cotton fabrics are the main factors that determine the thermo-mechanical properties of coated materials.

Thus, the storage modulus recorded in the case of D4 was high relative to uncoated cotton fabrics due to the rigidity induced by the silica coating, which constrains the motion of the cellulose chains. When TGIC is added to the nanosol composition, as in the case of D8, the storage modulus is further increased by about 30%. This can be explained only by a higher degree of crosslinking. This is sustained by the stability of the storage modulus as a function of temperature, which is also observed in the case of D8 when the signal change did not exceed 10% of the initial value at 200 °C, comparable to D4. In the latter case, the loss modulus is higher because the energy lost through viscous heating is dominant during the stressing process. This is a consequence of the organic chains generated by epoxy homopolymerization induced by NMI, which provide flexibility to the coated fabrics. A higher degree of crosslinking obtained due to the isocyanurate groups creates a broader loss factor curve. This suggests the existence of molecular relaxation and strong interactions at the interface, which reduces the mobility of the molecules and determines the loss factor values. The peak high is an important indication of the energy dissipated, and the higher values obtained in the case of D4 relative to D8 signify a poor interface between the coating and the fabric.

**Figure 11:**
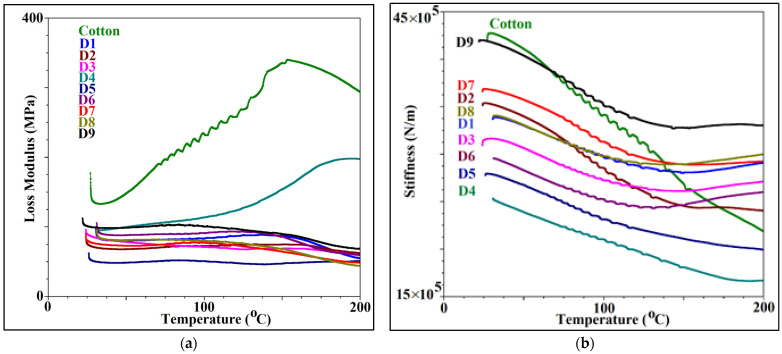
Loss Modulus (**a**) and stiffness (**b**) of the coated fabrics.

Flexibility and the level of the loss modulus can be connected, and therefore, the higher the loss modulus, the greater the flexibility of the coated textile materials. In the case of D4, not only is the value of the modulus at room temperature the highest among all of the samples analyzed and is the closest to that of the uncoated cotton fabric, but also its heating behavior is much more similar, as can be observed in Figure 11a. Obviously, the presence of GLYMO and MIM in the nanosol film-forming material leads to a more flexible coating due to the development of organic homopolymeric chains with crosslinking only in the inorganic area and fewer anchoring points with the structure of the textile material. Thus, the carbon chains in the GLYMO structure are responsible for the greater flexibility of the interface with the inorganic domains generated by silanols, while the oligomeric structures with ethylene oxide units give flexibility to the interface of the hybrid coating with the textile material. TGIC and BORAX are crosslinking agents that react both with the alcoholic groups belonging to the textile materials and to the oligomeric structures with ethylene oxide units giving rise to additional crosslinking points, which is manifested by a lower flexibility of the coatings, proven by the lower values of the loss modulus.

Stiffness is lower for D4 related to D8 due to an improved interfacial adhesion that can be ascribed to more hydrogen bond interactions between the hydroxyl groups of cellulose chains and of the sol–gel coating material generated during epoxy homopolymerization, as can be observed in Figure 11.

The effect of light on the stability of the coated materials can be estimated by the comparison of DMA characteristics of the coated fabrics before and after irradiation, as it can be seen from Table 3. Therefore, after the exposure of D4 to LED, an increased storage modulus of about 35% is observed, while the loss modulus is diminished by 25% when high and maximum temperatures are shifted to lower values, about 15°. When the same sample was exposed to xenon light, the stiffness was further increased, which suggests a crosslinking process.

From the data presented, it appears that the presence of GLYMO in the composition of nanosols has a beneficial effect on the thermo-mechanical properties, as observed in the case of D4. This was also reflected in the results obtained in the case of the other resistance properties of the photocatalytic xerogel coating, as it was seen in those exposed previously.

## 3. Conclusions

The hybrid silica gel coating materials containing TiO_2_-FeTCPc proposed by us for the functionalization of cellulosic textile supports represent a good solution from the perspective of the practical applications they can offer. This study shows that there are solutions for obtaining high photocatalytic efficiency through exposure to LED light (only visible light). In the attempt to obtain xerogel coatings resistant to wet treatments, several variants of nanosols were tried, but only the presence of GLYMO and the homopolymerization of epoxy groups in the presence of MIM led to obtaining convenient coatings on cotton. Both from the point of view of stiffness and from the point of view of photocatalytic efficiency in the MB degradation process, the option of crosslinking with BORAX is inappropriate, even if the resistance to wet treatments of photocatalytic cellulosic fabrics is better. The equilibrium solution found after the study is the one given by the existence of GLYMO, MIM, and TGIC in the photocatalytic nanosol. They ensure the very good compatibility of the photocatalytic xerogel film with the cellulosic textile material, maintaining a high effectiveness of the TiO_2_-FeTCPc, good resistance of the coating to light and wet treatments, and a convenient value of the coated material stiffness.

## 4. Materials and Methods

### 4.1. Materials

Silica hybrid nanosols were obtained using the following reagents: hydrochloric acid (0.1 N, HCl), ethanol (96%, EtOH), isopropyl alcohol (97%, iPrOH), and sodium tetraborate (99%, BORAX), were obtained from Chimreactiv, Bucharest, Romania; tetraethylortosilicate (98%, TEOS), 3-(2,3-epoxypropoxy)propyltrimethoxysilane (98%, GLYMO), octyltriethoxysilane (97%, OTES), phenyltriethoxysilane (98%, PTES), tetrahydrofuran (inhibitor-free, 99.9%, THF), 1-methylimidazole (99%, MIM), methylene blue (97%, MB), and tris(2,3-epoxypropyl) isocyanurate (TGIC) were obtained from Merck, Darmstadt, Germany and were used as they were received. Aeroxide P25 was procured from Evonik, city, Germany. The cellulose fabric used during the experiments was a plain woven scoured and chemically bleached 100% cotton fabric, with a specific weight of 106 g/m^2^, supplied by Matasea Romana SA, Cisnadie, Romania.

TiO_2_ sensitized with iron (III) phthalocyanine tetracarboxylic acid (FeTCPc) was obtained by us, as it was already described [25]. Briefly, in order to create a fine suspension of TiO_2_, 50 mL of isopropyl alcohol containing 0.2 mL of 25% (by weight) ammonia aqueous solution was combined with 1.9 g TiO_2_ powder (P25) and 0.05 g FeTCPC. The titanium dioxide nanoparticles that had been modified with FeTCPC were removed from the reaction mass after it had been heated under reflux for 5 h. They were then washed with de-ionized water, filtered, and dried in an oven at 120 °C for 10 h.

### 4.2. Methods

#### 4.2.1. Preparation of Photocatalytic Nanosols and the Pad–Dry–Cure Procedure

For an hour, at room temperature, a mixture containing 1.5 g TEOS, 1.7 g GLYMO, 0.65 mL water, 3.7 mL ethanol, and 0.7 mL of hydrochloric acid (0.1 N) was agitated. Then, a dispersion made of 0.45 g of TiO_2_-FeTCPc in 2 mL of isopropyl alcohol to which 0.01 g of MIM was added, was obtained by stirring for 10 min at a power of 150 W with an ultrasonic processor VCX 750 (SONICS Inc., Newtown, CT, USA). The liquid was kept warm and vigorously stirred before being used right away to impregnate the cellulose fabrics. Before being used, TGIC and/or BORAX were added to the nanosols, in the situations and in the quantities provided in Table 1.

By using the pad–dry–cure method, the photocatalytic nanosols with a content of 5% (by weight) TiO_2_ sensitized with FeTCPC were applied to a 2 g cotton fabric at a wet pick-up of 75 to 80%. The best impregnation conditions were found at a constant speed of 0.5 m/min and a pressure of 0.4 kg/cm^2^ on a horizontal laboratory padding mangle made by Ernst Bentz AG in Kanton Zurich, Dielsdorf, Switzerland. After four cycles of passing through the nanosol bath, the cotton materials were dried at room temperature. Later, in some cases of crosslinking with BORAX, the textile samples were subjected to impregnation under the same conditions with 100 mL of aqueous solution containing 25 g of BORAX and were then dried at room temperature. In order to accelerate and complete the crosslinking process without compromising the structure of the support fabric, all textile samples finished with nanosols were heated at 130 °C for one hour in a thermofixation oven.

#### 4.2.2. Characterization Methods and Equipment

The coated fabrics’ structural and morphological characteristics were assessed using the following techniques: Using a JASCO FT-IR 6300 instrument (Jasco Int. Co. Ltd., Tokyo, Japan) fitted with a Specac ATR Golden Gate (Specac Ltd., Orpington, UK) with KRS5 lens, measurements of the FTIR-ATR spectra of the nanosol-coated fabrics were performed in the 400–4000 cm^−1^ range (32 accumulations at a resolution of 4 cm^−1^). With a scanning electron microscope (TM4000Plus; HITACHI, Tokyo, Japan) operating at an accelerating voltage of 10 kV and magnifications up to 1800, images of the coated cotton samples were collected. The BET (Brunauer-Emmett-Teller) and BJH (Barrett-Joyner-Halenda) techniques were used to assess the hybrid materials’ porosity characteristics. Quantachrome’s Nova 2200e automated gas adsorption system (Quantachrome Instruments Corporate Drive, Boynton Beach, FL, USA) was used to measure the N_2_ adsorption-desorption isotherms at −196 °C and determine the specific surface area (SBET) and total pore volumes (Vtotal) of the samples that were previously degassed at 100 °C for three hours. Samples were subjected to X-ray diffraction (XRD) analysis using a Rigaku SmartLab X-ray diffractometer (Rigaku, Tokyo, Japan) equipped with a vertical goniometer in parallel beam geometry at ambient temperature, utilizing Cu K radiation (λ = 1.54056 Å). A Park Systems XE 100 microscope (Park Systems, Suwon, Republic of Korea) was used to conduct atomic force microscopy (AFM) in the noncontact mode. Six water contact angle (WCA) measurements for each sample were used to assess the hydrophobic qualities of the fabrics covered with nanocomposites using a CAM 200 (KSV Instruments, Helsinki, Finland) instrument equipped with an auto-dispenser and a high-resolution camera (Basler A602f, Basler, Ahrensburg, Germany). After 2 s from the impact of 6 µL droplets of deionized water, which were spread out on the surface of each sample, the water contact angle (WCA) was determined as the average of the six measurements in air at room temperature and ambient humidity. The following techniques were used to assess the optical characteristics of the nanosols deposited on the textiles: A V570 UV-VIS-NIR spectrophotometer equipped with a ILN-472 (150 mm) integrating sphere (Jasco Int. Co. Ltd., Tokyo, Japan) was used to measure the diffuse reflectance spectra and total color differences in the CIELAB system using a 10° standard observer and illuminant D65. Spectralon was used as the reference, and Spectra Manager I software (Jasco Int. Co. Ltd., Tokyo, Japan) was used to process the data. Finished cotton fabrics were tested using ISO 105-C06 [39] and ISO-105X12 [40], respectively, for color fastness to washing and rubbing. The four different sample types were cut into 5 × 5 cm pieces for these evaluations, and each piece was sewn between a cotton and a wool fabric. On Linitest testing equipment (Atlas, Rock Hill, IL, USA), the produced sandwiches were washed with an aqueous solution of 1% by weight sodium dodecyl sulfate at 60 °C for an hour. The samples were opened and left to air dry after being rinsed with hot and cold water.

Photodegradation experiments were performed using as a model contaminant methylene blue (MB) and UV–Vis spectroscopy to detect changes in the MB color intensity during the photocatalytical reactions. Methylene blue aqueous solution of 1 g/L concentration was spread in droplets onto the surface of the coated textile samples with subsequent drying in an oven for 2 h at 120 °C. Samples were irradiated using two LED projectors of 100 W each (Super Bright LEDs Inc., St. Louis, MO, USA), providing a warm white light (correlated color temperature 2700–3000 K). On the surface of the coated textile samples, the irradiance was measured with an LP 471 RAD radiometric probe for effective irradiance measurement in the spectral range 400–1050 nm, connected to a Delta OHM-HD 2302.0 Light-meter (Delta OHM Srl, Padova, Italy), the average value obtained being 30 ± 1 W/cm^2^. Similar samples were irradiated using parameters established according to ISO 105-B02 [41] (irradiance of 42 ± 2 W/cm^2^ in the range 300–400 nm) in an ATLAS-Xenotest 150S+ device (2200 W, Atlas Material Testing Solutions, Mount Prospect, IL, USA). The concentration of MB was determined during the photocatalytic tests from visible diffuse reflectance spectra, and the degradation efficiency (%) was measured from the most significant minimum of the reflectance spectrum using Formula (2):

DE = (*R*_t_ − *R*_0_)/*R*_0_ × 100 (2)

where DE is degradation efficiency (%), *R*_t_ represents the reflectance at various intervals of exposure to light, and *R*_0_ is the initial reflectance of the coating.

On specimens measuring 10 × 10 × 0.57 mm, dynamic mechanic analysis was carried out using a Q800 instrument (TA Instruments, New Castle, DE, USA) in the multi-frequencies-strain mode with a shear sandwich clamp, operated at a fixed frequency of 1 Hz, oscillation amplitude of 20 m, and temperature ramp of 3 °C/min, in air, from ambient temperature to 200 °C. Universal Analysis software (TA Instruments, New Castle, DE, USA) was used to process the data.

## Figures and Tables

**Table 1:** Nanosols film-forming compositions and after treatments performed during coatings deposition onto the cellulose fabrics.

Sample	TEOS [g]	GLYMO [g]	MIM [g]	TGIC [g]	BORAX [g]	TiO_2_-FeTCPc [g]
D1	1.5	1.7	-	0.4	-	0.45
D2	1.5	1.7	-	0.4	0.25 (subseq. stage)	0.45
D3	1.5	1.7	-	0.4	0.25	0.45
D4	1.5	1.7	0.01	-	-	0.45
D5	1.5	1.7	0.01	-	0.25 (subseq. stage)	0.45
D6	1.5	1.7	0.01	0.4	-	0.45
D7	1.5	1.7	0.01	0.4	0.25 (subseq. stage)	0.45
D8	1.5	1.7	0.01	0.4	-	0.45
D9	1.5	1.7	0.01	0.4	0.25	0.45

**Table 2:** Textural properties of the coated fabrics.

Sample	Surface Area [m^2^/g]	Pore Volume [cm^3^/g]	Pore Size [nm]
Cotton fabric	3.8 (±0.5)	0.004 (±0.001)	2.97 (±0.03)
D1	5.1 (±0.2)	0.007 (±0.002)	3.11 (±0.04)
D2	3.0 (±0.2)	0.004 (±0.001)	2.96 (±0.03)
D3	3.7 (±0.4)	0.004 (±0.001)	2.97 (±0.03)
D4	3.0 (±0.3)	0.004 (±0.001)	4.24 (±0.04)
D5	3.2 (±0.3)	0.004 (±0.001)	4.27 (±0.04)
D6	2.7 (±0.4)	0.004 (±0.001)	3.44 (±0.04)
D7	3.3 (±0.4)	0.004 (±0.001)	2.96 (±0.03)
D8	3.4 (±0.2)	0.005 (±0.001)	2.97 (±0.03)
D9	3.1 (±0.2)	0.005 (±0.001)	3.82 (±0.04)

**Table 3:** Thermo-mechanical properties of the coated fabrics.

Sample (30 °C)	Storage Modulus (G′) (MPa)	Stiffness (N/m)	Loss Modulus		Loss Factor	
			Temperature (°C)	(G″) (MPa)	Temperature (°C)	Tan δ
D4 initial	1332	2,530,980	191.09	197.3	193.24	0.2259
D4 LED	1804	3,487,350	175.07	145.6	176.61	0.1048
D4 xenon	2087	3,755,500	175.37	126.1	178.15	0.07197
D6 initial	1527	2,952,510	117.76	92.91	124.54	0.07339
D6 LED	1764	3,411,160	117.76	79.91	122.69	0.05239
D6 xenon	2061	3,847,290	130.7	71.92	138.71	0.04058
D8 initial	1756	3,395,640	94.65	80.03	106.98	0.05179
D8 LED	1820	3,578,840	102.05	81.96	107.28	0.05201
D8 xenon	1850	3,576,450	149.49	72.5	153.5	0.04735

## Data Availability

The data are not publicly available due to containing information that could compromise the privacy of the research participants.

## Supplementary Materials

The following supporting information can be downloaded at: https://www.mdpi.com/article/10.3390/gels9110860/s1, Scheme S1: Mechanism of GLYMO homopolymerization catalyzed by MIM; Figure S1: FTIR spectra of D4 exposed to light; Figure S2. X-ray diffraction pattern of all coated fabrics; Figure S3. Mechanism of charge and reactive species generation under LED light illumination.
