# Supplementary material for: Cellulose Fabrics Functionalized with Sol–Gel Photocatalytic Coatings Based on Iron (III) Phthalocyanine Tetracarboxylic Acids–TiO2–Silica Hybrids
Source: Gels. 2023 Oct 30;9(11):860. doi: 10.3390/gels9110860 (PMC10671179; doi:10.3390/gels9110860)
Supplement: Supplementary file 1 [file gels-09-00860-s001.zip › gels-2672025-supplementary.pdf]

[Supporting Information]

Cellulose Fabrics Functionalized with Sol-Gel Photocatalytic Coatings Based on Iron (III) Phthalocyanine Tetracarboxylic Acids-TiO_2-Silica Hybrids

Alina Raditoiu[1], Valentin Raditoiu [1,*], Monica Florentina Raduly [1], Augusta Raluca Gabor [1], Adriana Nicoleta Frone [1], Maria Grapin [1] and Mihai Anastasescu [2]

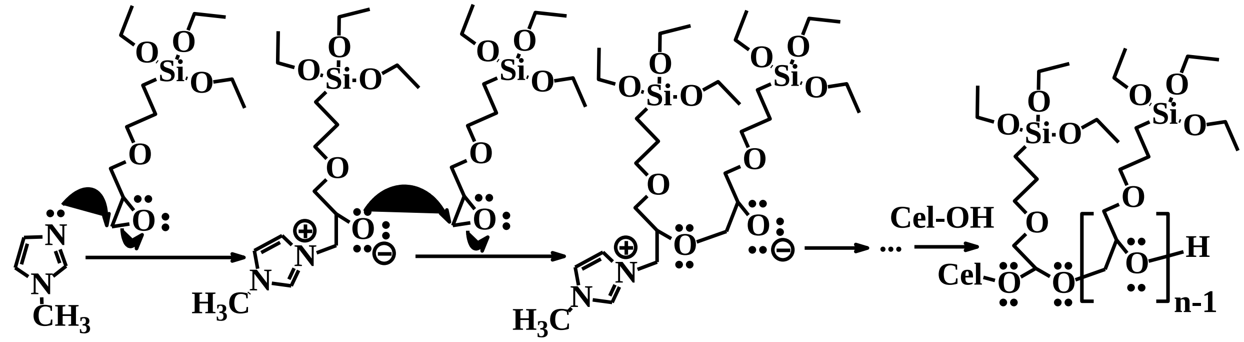

Scheme S1. Mechanism of GLYMO homopolymerization catalyzed by MIM

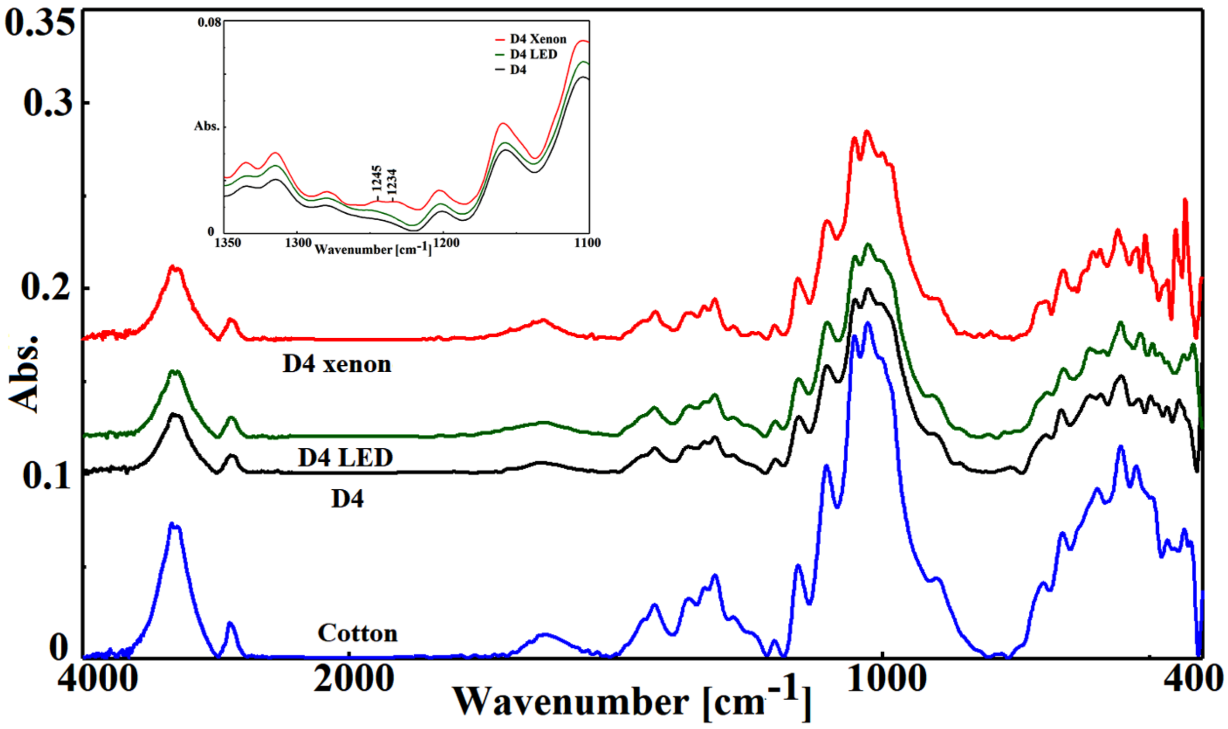

Figure S1. FTIR spectra of D4 exposed to light

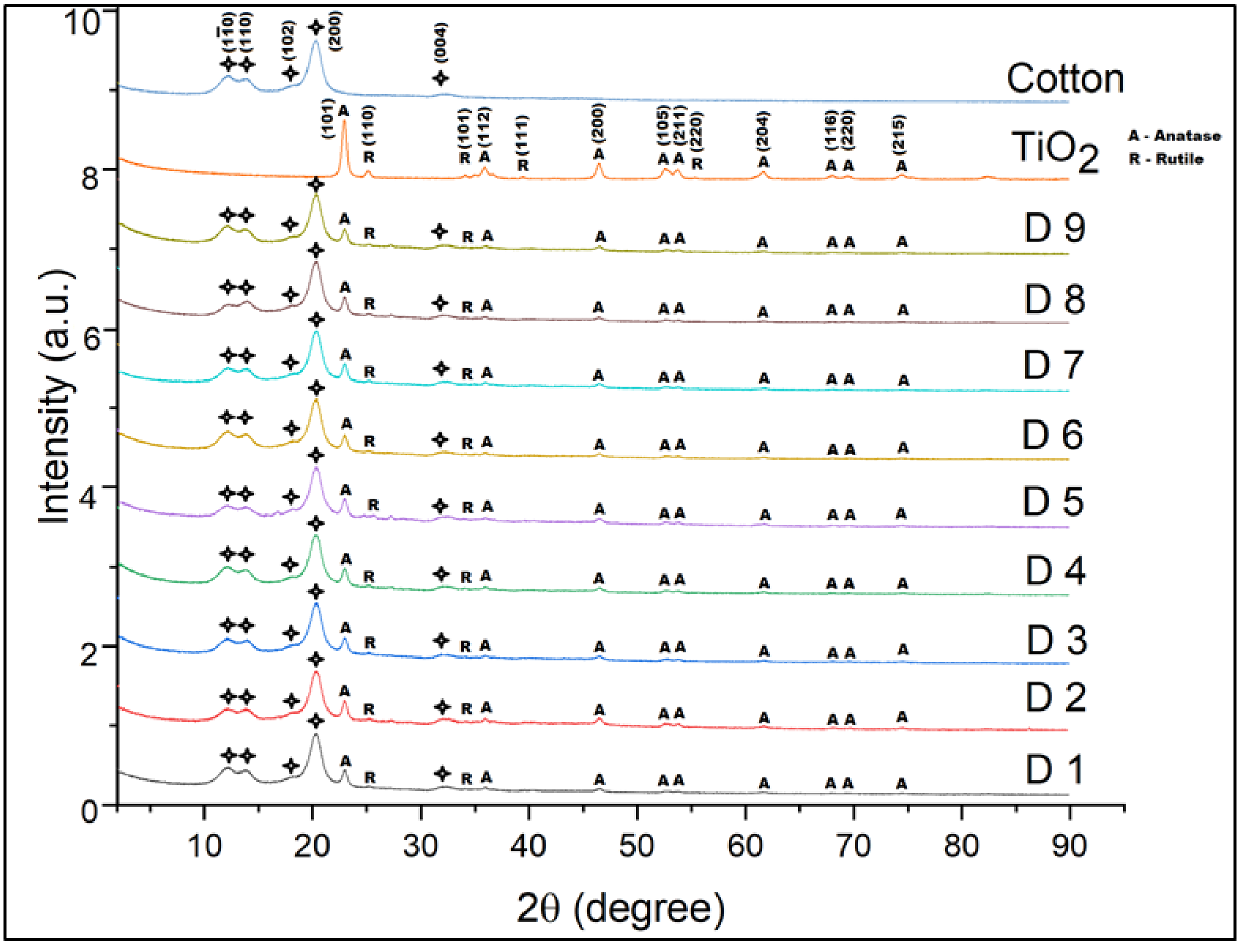

Figure S2. X-ray diffraction pattern of all coated fabrics

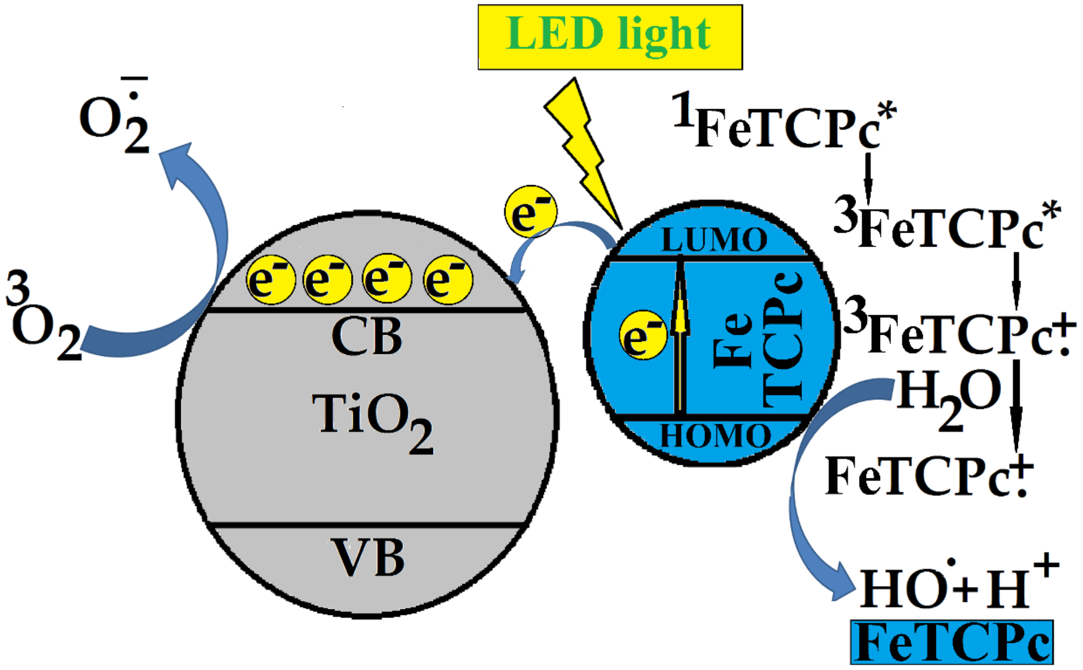

Figure S3. Mechanism of charge and reactive species generation under LED light illumination
